# Post-traumatic Stress Disorder as an Independent Risk Factor for Increased Opioid Use Following Carpal Tunnel Surgery

**DOI:** 10.1177/15589447231160207

**Published:** 2023-03-23

**Authors:** Nicholas L. Hudock, Sean A. Kshir, Kenneth F. Taylor

**Affiliations:** 1 Penn State Health Milton S. Hershey Medical Center, Hershey, PA, USA; 2 Penn State College of Medicine, Hershey, PA, USA

**Keywords:** carpal tunnel syndrome, nerve, diagnosis, pain, disability, research and health outcomes, outcomes, psychosocial, pain management, specialty

## Abstract

**Background:**

Carpal tunnel release (CTR) is one of the most common hand surgeries. Studies have highlighted a mental-physical connection to hand pathologies and psychological connections to postoperative pain burden. Post-traumatic stress disorder (PTSD) has been identified as a medical-psychological comorbidity like other mental health disorders such as generalized anxiety disorder (GAD). There remains a gap in the literature regarding PTSD as a comorbidity for hand surgeries, where there is this mental-physical connection. We hypothesize PTSD will be associated with increased risk of postoperative pain, evidenced by greater prevalence of opioid usage.

**Methods:**

The authors performed a retrospective analysis using the TriNetX Research Database. Patients who underwent elective CTR were identified within the database. Two groups were created and compared against individual controls: the first was identified based on the diagnosis of PTSD, and the second was identified based on the diagnosis of GAD. Cohorts were matched and opioid usage was compared postoperatively.

**Results:**

Patients with PTSD who underwent CTR were found to be at significantly increased risk of postoperative opioid use (*P* = .033) and more likely to present to the emergency department (ED) (*P* = .001). Patients with GAD were found to be significantly less likely to require postoperative opioids (*P* = .040).

**Conclusions:**

We found patients with PTSD to be at increased risk of opioid use and more likely to present to ED following CTR. Patients with GAD were found to be at decreased risk of opioid use after CTR. Owing to the independent significant risks not found in GAD, further research of postoperative pain in patients with PTSD is needed.

## Introduction

Post-traumatic stress disorder (PTSD) is a psychiatric condition that can develop after experiencing a potentially life-threatening event such as combat, sexual violence, natural disaster, or serious injury.^1^ A review of PTSD in the United States indicates a lifetime prevalence of 7.8% among civilians and illustrates that US combat veterans are up to 2 to 4 times more likely to suffer from PTSD than their civilian counterparts.^2^ Studies of mental health as a surgical comorbidity have classically focused on diseases such as general anxiety disorder (GAD), major depressive disorder, or the thought patterns associated with risks of poor surgical recovery.^3,4^ Thus, there is an unmet need to explore PTSD as an independent risk factor for surgical comorbidity.

Carpal tunnel release (CTR) is one of the most common hand surgeries with an estimated 400 000+ surgeries performed each year in the United States.^5^ Complications of CTR are uncommon but include general surgical risks, such as infection, and more procedure-specific complications, such as pain syndromes and treatment failures.^6^ Since the 1980s, psychiatrists have recognized the physical manifestations of mental illness and mental manifestations of physical illness.^7^ The introduction of case reports examining psychosomatic clenched fist syndrome has highlighted these unique mental-physical connections and conversion disorders among orthopedic hand surgery patients.^8^ Given the clinical volume and relative safety of carpal tunnel surgery in combination with the emerging recognition of mental-physical connections to the hand, determination of PTSD as an independent risk factor for postoperative CTR complications is of great interest for identifying at-risk patients and developing prevention and intervention strategies.

Our study aims to narrow the gap in the literature regarding PTSD as a surgical comorbidity by exploring the outcomes of patients undergoing CTR. We hypothesize PTSD will be associated with increased risk of postoperative pain, evidenced by greater prevalence of opioid usage. Furthermore, we suspect PTSD will be associated with greater opioid usage and increased risk of common complications of CTR compared with GAD. The results of this study may be useful in the determination of PTSD as an independent risk factor for increased postoperative pain and the identification of at-risk patients.

## Methods

A retrospective case-control database study was performed using TriNetX Research Network to query for patients who had undergone CTR defined through the Current Procedural Terminology codes. The TriNetX Global Collaborative Network was used. At the time of the study, this included 86 health care organizations and totaled more than 110 million patients over the 2002- 2022 period. TriNetX provides a deidentified research population derived from electronic medical records that permit query-based data extraction from medical claims including inpatient and outpatient encounters, as well as pharmacy records. The process by which the data are deidentified is attested to through a formal determination by a qualified expert as defined in Section §164.514(b)(1) of the Health Insurance Portability and Accountability Act (HIPAA) Privacy Rule.^9^ This formal determination by a qualified expert supersedes TriNetX’s waiver from the Western Institutional Review Board (IRB). Penn State has an agreement in place to access the TriNetX Research Network, and there was no funding associated with this study.

Two queries were performed: the first to compare patients who have a diagnosis of PTSD (International Classification of Diseases [ICD]10CM:F43.1) without GAD (ICD10CM:F41.1) who have undergone CTR (open approach: ICD10PCS:01N50ZZ, or percutaneous endoscopic approach: ICD10PCS:01N54ZZ) with a cohort of patients who have undergone CTR surgery who do not have a diagnosis of PTSD or GAD; and the second to compare patients who have a diagnosis of GAD without PTSD who have undergone CTR with a cohort of CTR patients who do not have a diagnosis of PTSD or GAD. In both queries, the results were not reviewed before propensity score matching by age, sex, ethnicity, diabetes, cardiovascular risk factors, opioid use, and additional substance abuse including nicotine dependence and alcohol abuse disorders.

The data were analyzed initially through TriNetX software (Maker) (Cambridge, MA), which uses JAVA, R, and Python programming languages. Measures of association were calculated, including risk ratios, risk differences with *t* tests, and odds ratios, along with 95% confidence interval (CI) for each, respectively. Additional table formation was performed using Microsoft Excel.

The primary objective of this study was to identify the risk of opioid use within 30 days postoperatively of patients with PTSD who have undergone elective CTR. The risk of opioid use was defined in this study by the presence of an opioid prescription. Secondary outcomes include 30-day risk of opioid use in patients with GAD who have undergone elective CTR, as well as 30-day risk of diagnosis of chronic pain syndrome, surgical site infection, sepsis, emergency department (ED) presentation, or development of delirium in patients with either PTSD or GAD who have undergone elective CTR.

## Results

A total of 1079 patients who were diagnosed with PTSD and had undergone CTR were identified. A total of 51 016 patients who had undergone CTR without a diagnosis of PTSD or GAD were identified. After propensity score matching, 1075 patients remained in both cohorts. The average age of patients with PTSD was 48.5 years, and the age of those without a diagnosis of PTSD was 48.4 years. Most patients were woman (60.2% and 58.7%) and white (66.1% and 66.6%) among the PTSD cohort and those without PTSD who had undergone CTR, respectively. Prior to CTR, 28.7% and 28.9% of PTSD and control cohort patients were using opioids, respectively. A full summary of characteristics, demographics, and comorbidities is summarized in Table 1.

**Table 1.** Demographics and Comorbidities of the PTSD Cohort.

Variables	Unmatched					Matched				
Characteristic name	PTSD	% of cohort	No PTSD	% of cohort	*P* value	PTSD	% of cohort	No PTSD	% of cohort	*P* value
No. of patients	1079		51 016			1075		1075		
Age at index	48.5 ± 13.8		55.4 ± 15.6		<.001	48.5 ± 13.9		48.4 ± 14.2		.818
Male	430	39.90	20 021	39.20	.686	428	39.80	444	41.30	.482
Female	649	60.10	30 994	60.80	.687	647	60.20	631	58.70	.482
Non-Hispanic or Latino	772	71.50	25 251	49.50	<.001	769	71.50	809	75.30	.051
Hispanic or Latino	45	4.20	1500	2.90	.018	45	4.20	49	4.60	.673
Unknown ethnicity	262	24.30	24 265	47.60	<.001	261	24.30	217	20.20	.022
White	713	66.10	28 724	56.30	<.001	711	66.10	716	66.60	.819
Black or African American	176	16.30	5300	10.40	<.001	175	16.30	188	17.50	.454
Asian	10	0.90	414	0.80	.677	10	0.90	10	0.90	1
American Indian or Alaska Native	10	0.90	187	0.40	.003	10	0.90	10	0.90	1
Unknown race	172	15.90	16 360	32.10	<.001	171	15.90	161	15.00	.551
Opioid analgesics	312	28.90	7125	14.00	<.001	308	28.70	311	28.90	.886
Nicotine dependence	115	10.70	1496	2.90	<.001	111	10.30	102	9.50	.516
Alcohol-related disorders	44	4.10	277	0.50	<.001	40	3.70	32	3.00	.338
Diabetes mellitus	127	11.80	2611	5.10	<.001	123	11.40	116	10.80	.631
Hypertensive diseases	195	18.10	5653	11.10	<.001	191	17.80	184	17.10	.691
Hyperlipidemia, unspecified	73	6.80	2054	4.00	<.001	72	6.70	62	5.80	.372
Heart failure	25	2.30	418	0.80	<.001	23	2.10	15	1.40	.19
Other venous embolism and thrombosis	15	1.40	263	0.50	<.001	15	1.40	10	0.90	.314
Peripheral vascular disease, unspecified	10	0.90	180	0.40	.002	10	0.90	10	0.90	1
Acute myocardial infarction	10	0.90	164	0.30	.001	10	0.90	10	0.90	1
Atrial fibrillation and flutter	12	1.10	610	1.20	.803	12	1.10	10	0.90	.668

*Note.* Age, ethnicity, race, preoperative opioid usage, and medical and surgical comorbidities among PTSD and control cohorts both before and after propensity matching. After matching, there were no significant differences among cohorts in the variables explored. PTSD = post-traumatic stress disorder.

A total of 3131 patients were identified who were diagnosed with GAD and underwent CTR; 51 015 patients were identified who had undergone CTR without a diagnosis of PTSD or GAD. After propensity matching, 3121 patients remained in each cohort. The average age of patients with a diagnosis of GAD was 56.2 years. The average age of those without a diagnosis of GAD was 55.9 years. Again, most patients were woman (72.5% and 71.8%) and white (82.4% and 82.8%) in the GAD cohort and control cohort, respectively. Prior to CTR, 24.0% and 23.4% of GAD and control cohort patients were using opioids, respectively. A full summary of characteristics, demographics, and comorbidities is summarized in Table 2.

**Table 2.** Demographics and Comorbidities of the GAD Cohort.

Variables	Unmatched					Matched				
Characteristic name	GAD	% of cohort	No GAD	% of cohort	*P* value	GAD	% of cohort	No GAD	% of cohort	*P* value
No. of patients	3131		51 015			3121		3121		
Age at index	56.2 ± 14.9		55.4 ± 15.6		.002	56.2 ± 14.8		55.9 ± 15.0		.512
Male	859	27.40	20 021	39.20	<.001	858	27.50	880	28.20	.534
Female	2272	72.60	30 993	60.80	<.001	2263	72.50	2241	71.80	.534
Non-Hispanic or Latino	2497	79.80	25 250	49.50	<.001	2487	79.70	2522	80.80	.266
Hispanic or Latino	90	2.90	1500	2.90	.832	90	2.90	105	3.40	.275
Unknown ethnicity	544	17.40	24 265	47.60	<.001	544	17.40	494	15.80	.089
White	2581	82.40	28 723	56.30	<.001	2571	82.40	2584	82.80	.664
Black or African American	281	9.00	5300	10.40	.012	281	9.00	284	9.10	.895
Asian	19	0.60	414	0.80	.212	19	0.60	21	0.70	.751
American Indian or Alaska Native	10	0.30	187	0.40	.67	10	0.30	13	0.40	.531
Unknown race	241	7.70	16 360	32.10	<.001	241	7.70	217	7.00	.244
Opioid analgesics	757	24.20	7125	14.00	<.001	750	24.00	731	23.40	.572
Nicotine dependence	178	5.70	1496	2.90	<.001	175	5.60	146	4.70	.097
Alcohol-related disorders	57	1.80	277	0.50	<.001	55	1.80	56	1.80	.924
Diabetes mellitus	386	12.30	2611	5.10	<.001	379	12.10	357	11.40	.388
Hypertensive diseases	702	22.40	5653	11.10	<.001	693	22.20	671	21.50	.5
Hyperlipidemia, unspecified	299	9.50	2054	4.00	<.001	293	9.40	269	8.60	.289
Heart failure	116	3.70	418	0.80	<.001	106	3.40	91	2.90	.277
Other venous embolism and thrombosis	58	1.90	263	0.50	<.001	53	1.70	45	1.40	.415
Peripheral vascular disease, unspecified	39	1.20	180	0.40	<.001	35	1.10	27	0.90	.307
Acute myocardial infarction	39	1.20	164	0.30	<.001	34	1.10	31	1.00	.708
Atrial fibrillation and flutter	115	3.70	610	1.20	<.001	108	3.50	82	2.60	.055

*Note.* Age, ethnicity, race, preoperative opioid usage, and medical and surgical comorbidities among GAD and control cohorts both before and after propensity matching. After matching, there were no significant differences among cohorts in the variables explored. GAD = generalized anxiety disorder.

**Figure 1.**
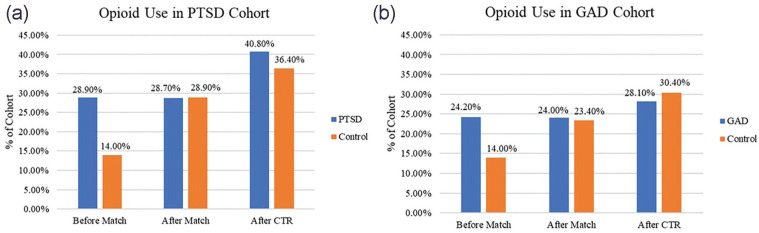
(a, b) Proportion of cohorts’ use of opioid medication. *Note.* Proportion of cohorts using opioid medication preoperatively, preoperatively after cohort balancing, and postoperatively among balanced cohorts. The x-axis highlights these groups, and the y-axis represents the percent of cohort. Values of postoperative opioid use without balancing were not provided by TriNetX and thus omitted. Proportions were used as PTSD and GAD cohorts differed in sample size. PTSD = post-traumatic stress disorder; GAD = generalized anxiety disorder; CTR = carpal tunnel release.

Outcomes were measured within 30 days after CTR. After CTR, 40.8% of patients with PTSD used opioids, and risk of opioid use was significantly increased compared with the healthy cohort (risk difference [RD]: 0.045; 95% CI, 0.004-0.086; *P* = .033) (Figure 1). Among the PTSD cohort, the risk of ED presentation was also significantly increased (RD: 0.032; 95% CI, 0.014-0.050; *P* = .001). Post-traumatic stress disorder was not found to significantly increase the risk of diagnosis of a chronic pain syndrome, surgical site infection, development of sepsis, or postoperative delirium. Those with GAD were found to be at significantly increased risk of diagnosis of a chronic pain syndrome (RD: 0.012; 95% CI, 0.007-0.017; *P* < .001), ED presentation (RD: 0.033; 95% CI, 0.023-0.044; *P* < .001), and development of postoperative delirium (RD: 0.006; 95% CI, 0.001-0.010; *P* = .011). Interestingly, patients with GAD were found to be significantly less likely to use postoperative opioids (RD: −0.024; 95% CI, −0.04 to, −0.001; *P* = .040); only 28.1% of patients used opioids within a month of surgery compared with the 30.4% of the control. Generalized anxiety disorder was not found to significantly increase the risk of surgical site infection or development of sepsis. A full summary of outcomes, risk, and significance can be found in Table 3.

**Table 3.** Outcomes Following Elective Carpal Tunnel Release.

Variables	Matched cohorts							Matched cohorts						
	PTSD		No PTSD		Risk difference	95% CI	*P* value	GAD		No GAD		Risk difference	95% CI	*P* value
	No. of patients with outcome	Risk	No. of patients with outcome	Risk				No. of patients with outcome	Risk	No. of patients with outcome	Risk			
Postoperative opioid use	439	0.408	391	0.364	0.045	0.004 to 0.086	.**033**	876	0.281	950	0.304	−0.024	−0.046 to −0.001	.**040**
Chronic pain syndrome	16	0.015	10	0.009	0.006	−0.004 to 0.015	.236	47	0.015	10	0.003	0.012	0.007 to 0.017	**<.001**
Surgical site infection	11	0.010	10	0.009	0.001	−0.007 to 0.009	.826	15	0.005	27	0.009	−0.004	−0.008 to 0.000	.063
Sepsis	11	0.010	10	0.009	0.001	−0.007 to 0.009	.826	35	0.011	32	0.010	0.001	−0.004 to 0.006	.713
ED presentation	69	0.064	35	0.033	0.032	0.014 to 0.050	.**001**	203	0.065	99	0.032	0.033	0.023 to 0.044	**<.001**
Postoperative delirium	18	0.017	10	0.009	0.007	−0.002 to 0.017	.128	34	0.011	16	0.005	0.006	0.001 to 0.010	.**011**

*Note*. Outcomes within 30 days of CTR. Shown are the number of patients of each cohort with the outcome or diagnosis, risk, risk difference with CI, and significance of difference. As mentioned in the “Discussion” section, one limitation of TriNetX is the use of a minimum patient sample of 10; for concerns of patient privacy, if a population is 1 or greater but less than 10, the population is counted as 10. PTSD = post-traumatic stress disorder; CI = confidence interval; GAD = generalized anxiety disorder; ED = emergency department; CTR = carpal tunnel release.

All bolded values significance of *p*<0.05.

## Discussion

This retrospective national database study represents a previously undescribed analysis of the surgical comorbidity of PTSD. We hypothesized patients with PTSD will be associated with an increased risk of postoperative pain as evidenced by greater prevalence of opioid usage, even in comparison with cohorts suffering mental health disorders such as GAD. This study found patients with PTSD are significantly more likely to require opioid medications following CTR (RD: 0.045; *P* = .033). This contrasts with patients with GAD who were found to require less opioids (RD: −0.024; *P* = .040). Furthermore, we suspected patients with PTSD would be at an increased risk of common complications of CTR comparable to other mental health disorders. Both patients with PTSD and GAD are significantly more likely to present to the ED within a month following CTR. Those with GAD are at an increased risk of delirium and diagnosis of chronic pain syndrome.

Studies of patients with PTSD have highlighted a heightened pain prevalence and pain response of this population. Not only has PTSD been correlated to increased experiences of chronic pain, but perception of pain was found to be more intense than controls of both healthy individuals and those with anxiety.^10,11^ In response to this increased pain, patients with PTSD, depression, anxiety, or catastrophic thinking were significantly more likely to be using opioid medications 1 to 2 months after surgery regardless of the injury severity or fracture site.^12^ The findings of increased opioid use among patient cohorts with anxiety are contradictory to our findings. One possible cause is our preoperative opioid usage cohort balancing. This has the possibility to artificially select for a greater proportion of patients from the “healthy” cohort with pain comorbidities. In an examination of minor hand surgeries, Vranceanu et al^13^ reported patients with greater pain intensity were correlated to pain catastrophizing but not necessarily hand disability. In studies of preoperative and postoperative pain, psychosocial factors continuously emerge as independent risks for increased pain and slowed recovery.

Despite the invasive and traumatic nature of surgery, few studies have assessed PTSD as a surgical comorbidity. Hudetz et al^14^ found decreased cognitive recovery after coronary artery surgery among veterans with PTSD, whereas Ikossi et al^15^ did not find PTSD to be a contraindication to gastric bypass surgery as patients had similar outcomes even with fluctuation in postoperative symptoms. While we found no evidence of infectious comorbidity to CTR in patients with PTSD or GAD, both were found to be at greater risk of presenting to the ED. The traditional model of illness assumes a direct relationship between trauma of surgery and pain or risk. Evidence suggests that in cases of mental comorbidities, minor traumas still have the potential to cause overinterpretation of nociception, likely leading to seeking medical attention.^16^ Even with these limited data focused on PTSD, the results suggest patients with PTSD may demonstrate increased difficulty in recovery.

A strength of our study is the use of a national database. This provided the ability to screen through millions of patients and achieve a sample size sufficient to propensity match cohorts based on demographics, preoperative opioid usage, and medical and surgical risk factors. For this reason, the use of TriNetX is accepted in current literature, including in studies using patient records and prescription usage.^17^ Unfortunately, use of this database is not without limitations. The predominant limitation is the lack of patient-level data. TriNetX operates using electronic health records, pharmacy records, and insurance billing data, detecting the presence or absence of medical, surgical, and prescription codes. To maintain HIPAA compliance and IRB exemption, these data are deidentified, and researchers are unable to access the exact relation between variables. We are not able to say with certainty the exact cause of patient’s postoperative findings, including opioid usage, ED presentation, delirium, or diagnosis of chronic pain. Findings of patients with anxiety experiencing increased risk of postoperative delirium have been reported.^18,19^ However, when we examine variables like “diagnosis of chronic pain syndrome,” we are finding the code for a diagnosis of chronic pain, but do not feel certain reporting this is necessarily in relation to the CTR. Our group elected to include this variable to assess the risk of pre-existing chronic pain conditions, given significant proportions of cohorts were on baseline opioid analgesics. We are further unable to determine the exact quantities of patient’s analgesic usage, both prescribed and taken. Finally, for patient privacy, if a population contains 1 or more but less than 10 patients, it will be counted as “10” patients; this has the possibility to interfere with significance of outcomes noted among the PTSD cohort. As shown in Figure 1, few patients of the control cohort had postoperative complications and were thus listed as “10.” The use of this database provides compelling evidence for further research examining patient-level data and allowing for the opportunity to report exact causation of postoperative opioid usage.

## Conclusions

Post-traumatic stress disorder was found to increase the risk of postoperative opioid use. In addition, PTSD increased the risk of presentation to the ED following CTR. Increased postoperative opioid use was not found in GAD. Due to the significant risks of increased postoperative pain, not found in a comparison mental health disorder, further research of postoperative pain in patients with PTSD is necessary.
